# Identification of Genomic Alterations in Thai Patients With Colorectal Cancer Using Next-Generation Sequencing-Based Multigene Cancer Panel

**DOI:** 10.7759/cureus.39067

**Published:** 2023-05-16

**Authors:** Worapoj Jinda, Hathaiwan Moungthard, Chanin Limwongse, Manop Pithukpakorn, Pensri Saelee, Nareerat Pokkasup, Saipan Khunpukdee, Suchitraporn Sukthaworn, Jaruphan Jumpasri

**Affiliations:** 1 Division of Research and Technology Assessment, National Cancer Institute, Bangkok, THA; 2 Division of Gastrointestinal and Liver Clinic, National Cancer Institute, Bangkok, THA; 3 Division of Medical Genetics, Department of Medicine, Faculty of Medicine, Siriraj Hospital, Mahidol University, Bangkok, THA; 4 Division of Policy and Medical Strategy Development, National Cancer Institute, Bangkok, THA

**Keywords:** pathogenic gene, cancer susceptibility genes, multigene cancer panel, next generation sequencing, colorectal cancer

## Abstract

Introduction

Colorectal cancer (CRC) is one of the leading causes of death and illness in the general population. Although the incidence of CRC is steadily decreasing worldwide, it is being diagnosed more in individuals under 50 years of age. Multiple disease-causing variants have been reported to be involved in the development of CRC. This study aimed to investigate the molecular and clinical characteristics of Thai patients with CRC.

Methods

NGS-based multigene cancer panel testing was performed on 21 unrelated patients. Target enrichment was performed using a custom-designed Ion AmpliSeq on-demand panel. Thirty-six genes associated with CRC and other cancer were analyzed for variant detection.

Results

Sixteen variants (five nonsense, eight missense, two deletions, and one duplication) in nine genes were identified in 12 patients. Eight (66.7%) patients harboring disease-causing deleterious variants in genes *APC*, *ATM*, *BRCA2*, *MSH2*, and *MUTYH*. One of the eight patients also carried additional heterozygous variants in genes *ATM*, *BMPR1A*, and *MUTYH*. In addition, four patients carried variants of uncertain significance in genes *APC*, *MLH1*, *MSH2*, *STK11*, and *TP53*. Among all detected genes, *APC* was the most frequent causative gene observed in CRC patients, which is consistent with previous reports.

Conclusion

This study demonstrated the comprehensive molecular and clinical characterization of CRC patients. These findings showed the benefits of using multigene cancer panel sequencing for pathogenic gene detection and showed the prevalence of genetic aberrations in Thai patients with CRC.

## Introduction

Colorectal cancer (CRC) is the third most commonly diagnosed cancer worldwide and the second cancer-related cause of mortality following lung cancer [1]. The most common age for individuals with CRC diagnosis is over 50 years old. Although the incidence of CRC has been steadily declining over the past few decades, the tendency of detection of early-onset CRC patients (under the age of 50) is increasing [2]. Data from the population-based cancer registry in Thailand have reported that CRC ranks as the third most prevalent cancer in males and as the second most prevalent cancer in females with an estimated 8,658 and 7,281 new cases, respectively, and there were estimated 4,781 deaths from this disease in an annual report 2017 [3,4].

Most early-stage CRCs are asymptomatic. As a result, CRC patients are often not diagnosed until they have reached an advanced stage of disease. Therefore, early detection has a significant impact on prognosis, treatment, and reducing its mortality. Due to the diversity of genes implicated in this disease, it is important to select a method that can screen for many genes at once.

Next-generation sequencing (NGS) is a powerful tool for detecting genetic alterations associated with various cancers. Advances in the development of this technology allow us to select the appropriate approach for cancer genetic testing. One of the NGS platforms is the sequencing of selected target genes according to the cancer relevance, creating a multigene panel that are cheaper and more cost-effective. It also provides molecular profiles of personalized cancer therapies and treatments. In the recent literature review, a multigene panel, including cancer susceptibility genes, has been used extensively in screening for patients with CRC [5-11].

The identification of a germline pathogenic variant in a known hereditary cancer-predisposing gene has an important implication for both patients and their family members because of its relevance in both clinical management according to a gene-specific approach for treatment and family planning. In this study, we proceeded to examine the prevalence of germline alterations with NGS-based targeted gene panel sequencing which included 36 genes associated with both known CRC-predisposing genes and other cancer susceptibility genes in 21 Thai patients with CRC.

## Materials and methods

Patients

Twenty-one unrelated Thai patients with primary CRC which consists of six (28.6%) cases with colon polyposis and 15 (71.4%) cases with only CRC. These patients who were older than 18 and had undergone surgery, chemotherapy, or radiotherapy were evaluated at the National Cancer Institute (NCI), Thailand, and recruited in this study. Genetic/familial high-risk evaluation of CRC was performed according to the 2019 National Comprehensive Cancer Network (NCCN) guideline. Their histopathological demographics were collected such as age at diagnosis, tumor site, staging, histologic grade, histologic type as well as metastasis. A detailed family history and pedigree chart were created. All patients consist of 12 males and nine females, with a median age at diagnosis of 38 years (ranging from 28 to 50 years). This study was conducted in accordance with the guidelines on Good Clinical Practice and the declaration of Helsinki and was approved by the Institutional Review Board of the NCI (IRB No. 64007) under the collaboration of the Molecular Genetics Laboratory, Siriraj Genomics, Office of the Dean, Faculty of Medicine Siriraj Hospital, Mahidol University, Bangkok, Thailand.

Peripheral blood samples

For the germline alterations analysis. Eighteen milliliters of peripheral blood were collected from the patient after obtaining written consent and sent to the Molecular Genetics Laboratory, Siriraj Genomics.

NGS-based multigene cancer panel testing

Briefly, the genomic DNA was extracted using Qiagen DNeasy DNA Isolation Kit (Hilden, Germany). Genetic analysis was performed using the NGS. Target enrichment was performed using a custom-designed Ion AmpliSeq on-demand panel (Ion Torrent S5 XL, Thermo Fisher Scientific, USA). The panel consists of 36 genes associated with CRC and other cancer (*APC, ATM, AXIN2, BARD1, BMPR1A, BRCA1, BRCA2, BRIP1, CDH1, CDK4, CDKN2A, CHEK2, EPCAM, FANCC, MLH1, MSH2, MSH3, MSH6, MUTYH, NBN, NF1, NTHL1, PALB2, PMS2, POLD1, POLE, PTEN, RAD50, RAD51C, RAD51D, RECQL, SMAD4, STK11, TP53, VHL*, and *XRCC2*) [12]. All variants passing filter criteria and copy number variants were validated with Sanger sequencing (the primer sequences for each gene used in this study are available upon request) and Multiplex Ligation-dependent Probe Amplification (MLPA) kits from MRC-Holland (Amsterdam, The Netherlands), respectively. The variants were interpreted according to the “Standards and Guidelines for the Interpretation of Sequence Variants” by the ACMG/AMP 2015, which are categorized into five different classes: pathogenic (P), likely pathogenic (LP), variant of unknown significance (VUS), likely benign (LB), and benign (B) [13]. LB and B variants were interpreted as negative. In families with identified variants, further target testing of such variants was performed in additional affected family members when available.

Protein structure and functional prediction

The protein structure analysis resulting from missense variants was predicted using the freely available web service HOPE (Have Your Protein Explained) (https://www3.cmbi.umcn.nl/hope/) [14].

## Results

**Figure 1:**
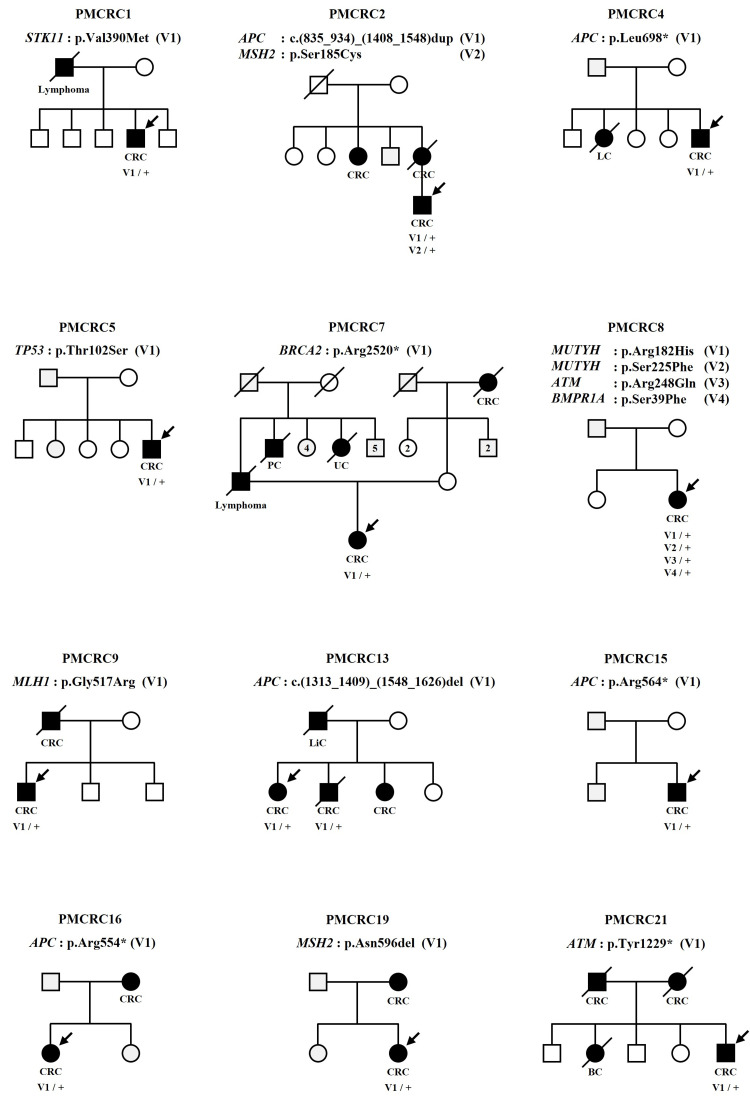
Pedigrees and genotypes of the CRC families. Genotype data are presented below the patient and members, where applicable, of their family. Filled symbols with an arrow indicate the probands. Squares: males. Circles: females. Slashed: decreased family members. Normal alleles are indicated by ‘+’. Variants are indicated by “V”. The number in the square represents the number of family members. CRC: colorectal cancer. LC: lung cancer. PC: pancreatic cancer. UC: uterine cancer. LiC: liver cancer. BC: bone cancer.

A total of 21 unrelated Thai patients with CRC were recruited in this study, consisting of 12 males and nine females. A multigene cancer panel was performed to identify the disease-causing variants in patients. According to variant analysis in 36 genes, a total of 16 different unique variants (five nonsense, eight missense, two deletions, and one duplication) were detected in 9/36 genes affected (25%). The deleterious P/LP variants were detected in eight out of 21 patients (38.1%). A summary of the clinical characteristics, the variants identified in this study, and family pedigrees are shown in Tables 1, 2, and Figure 1, respectively.

**Table 1:** Clinical information of patients in this study NA: not available

Sample no.	Gender	Age at diagnosis	Tumor Site	Staging	Histologic Grade	Histologic Type	Polyposis	Metastasis
PMCRC1	Male	47	Sigmoid colon	III	Moderately differentiated	Adenocarcinoma	None	No metastasis
PMCRC2	Male	29	Sigmoid colon	III	Moderately differentiated	Adenocarcinoma	Multiple	No metastasis
PMCRC3	Male	33	Rectum	IV	Moderately differentiated	Adenocarcinoma	None	Lung
PMCRC4	Male	46	Rectum	IV	Well differentiated	Adenocarcinoma	Multiple	Liver
PMCRC5	Male	47	Rectum	I	Well differentiated	Adenocarcinoma	None	No metastasis
PMCRC6	Male	39	Rectum	IV	NA	Adenocarcinoma	Multiple	Liver
PMCRC7	Female	33	Cecum to ascending colon	II	Moderately differentiated	Adenocarcinoma	None	No metastasis
PMCRC8	Female	32	Rectum	III	NA	Adenocarcinoma	None	No metastasis
PMCRC9	Male	38	Rectum	I	Well differentiated	Adenocarcinoma	None	No metastasis
PMCRC10	Male	46	Rectum	III	Moderately differentiated	Adenocarcinoma	None	No metastasis
PMCRC11	Female	32	Rectum	II	Moderately differentiated	Adenocarcinoma	None	No metastasis
PMCRC12	Female	37	Rectosigmoid colon	III	Well differentiated	Adenocarcinoma	None	No metastasis
PMCRC13	Female	40	Transverse colon	IV	Well differentiated	Adenocarcinoma	Multiple	NA
PMCRC13_ younger brother	Male	40	Rectum	IV	Well differentiated	Adenocarcinoma	Multiple	Bladder
PMCRC14	Male	49	Rectum	IIA	Moderately differentiated	Adenocarcinoma	None	No metastasis
PMCRC15	Male	28	Rectum	IV	Well differentiated	Adenocarcinoma	Multiple	Lung, Liver
PMCRC16	Female	30	Rectum	IV	Well differentiated	Adenocarcinoma	None	Lung
PMCRC17	Male	36	Ascending colon	IV	Well differentiated	Adenocarcinoma	None	Liver
PMCRC18	Female	33	Rectum	IV	Well differentiated	Adenocarcinoma	None	Lung
PMCRC19	Female	30	Descending colon	IV	Moderately differentiated	Adenocarcinoma	None	Lung
PMCRC20	Female	50	Ascending to sigmoid colon	-	Hyperplastic polyp	Tubular adenoma	Multiple	-
PMCRC21	Male	36	Transverse colon	III	Moderately differentiated	Adenocarcinoma	None	No metastasis

**Table 2:** Variants identified in the CRC patients Nucleotide numbering reflects cDNA numbering with position 1 corresponding to the A of the ATG translation initiation codon in the reference sequence, according to journal guidelines (provided in the public domain by The Human Genome Variation Society, http://www.hgvs.org/mutnomen). The initiation codon is codon 1. The nucleotide position of each gene was based on these GenBank cDNAs (accession number): *APC* (NM_038.5), *ATM* (NM_051.3), *BMPR1A* (NM_4329.2), *BRCA2* (NM_059.3),* MLH1* (NM_249.3), *MSH2* (NM_251.2), *MUTYH* (NM_1128425.1), *STK11* (NM_455.4), *TP53* (NM_546.5). The asterisk (*) indicates a stop codon predicted to terminate translation.

Patient ID	Gene	Inheritance Model	State	cDNA Change	Amino Acid Change	ClinVar Interpretation (Variation ID)	dbSNP rs ID	Variant Interpretation
PMCRC1	STK11	Dominant	Heterozygous	c.1168G>A	p.Val390Met	Uncertain Significance (142283)	rs374078532	VUS
PMCRC2	MSH2	Dominant	Heterozygous	c.554C>G	p.Ser185Cys	-	rs878853819	VUS
	APC	Dominant	Heterozygous	c.(835_934)_(1408_1548)dup (exon 10-11 duplication)	-	-	-	VUS
PMCRC4	APC	Dominant	Heterozygous	c.2093T>G	p.Leu698*	Pathogenic (433631)	rs137854582	Pathogenic
PMCRC5	TP53	Dominant	Heterozygous	c.304A>T	p.Thr102Ser	Uncertain Significance (582041)	rs1567555968	VUS
PMCRC7	BRCA2	Dominant	Heterozygous	c.7558C>T	p.Arg2520*	Pathogenic (52353)	rs80358981	Pathogenic
PMCRC8	MUTYH	Recessive	Heterozygous	c.545G>A	p.Arg182His	Pathogenic (182689)	rs143353451	Pathogenic
	MUTYH	Recessive	Heterozygous	c.674C>T	p.Ser225Phe	-	-	VUS
	ATM	Dominant	Heterozygous	c.743G>A	p.Arg248Gln	Uncertain Significance (479015)	rs769166447	VUS
	BMPR1A	Dominant	Heterozygous	c.116C>T	p.Ser39Phe	Uncertain Significance (230945)	rs876658859	VUS
PMCRC9	MLH1	Dominant	Heterozygous	c.1549G>A	p.Gly517Arg	Uncertain Significance (1404870)	-	VUS
PMCRC13	APC	Dominant	Heterozygous	c.(1313_1409)_(1548_1626)del	exon 12 deletion	-	-	Pathogenic
PMCRC15	APC	Dominant	Heterozygous	c.1690C>T	p.Arg564*	Pathogenic (808)	rs137854574	Pathogenic
PMCRC16	APC	Dominant	Heterozygous	c.1660C>T	p.Arg554*	Pathogenic (807)	rs137854573	Pathogenic
PMCRC19	MSH2	Dominant	Heterozygous	c.1786_1788delAAT	p.Asn596del	Pathogenic (1757)	rs63749831	Pathogenic
PMCRC21	ATM	Dominant	Heterozygous	c.3687C>G	p.Tyr1229*	-	-	Likely Pathogenic

Potentially pathogenic variants

Among all patients with detectable P/LP variants, *APC* accounted for four patients. In the meanwhile, the P/LP variants were also identified in other genes (*ATM, BRCA2, MSH2*, and *MUTYH*) that were found in four patients (Table 2).

Deleterious truncating variants (p.Arg554*, p.Arg564*, and Leu698*) in *APC* gene were observed in three patients (PMCRC16, PMCRC15, and PMCRC4), respectively. These three variants have been previously reported in the ClinVar database as P variants. In the PMCRC13 patient, a heterozygous alteration (c.(1313_1409)_(1548_1626)del) that causes deletion of exon 12 of the *APC* gene was detected by MLPA analysis. This variant has not been previously reported in any database. Interestingly, the same variant was also detected in her affected brother (Figure 1). According to the ACMG guidelines, this putative loss of function variant is classified as P.

In the patient, PMCRC19, a heterozygous three‐base deletion variant (c.1786_1788delAAT) was identified in the *MSH2* gene. This variant causes in-frame single amino acid deletion (p.Asn596del) in the DNA mismatch repair protein MSH2. It was reported as P in both the ClinVar (Variation ID: 1757) and the InSiGHT database (http://insight-database.org/), which has been shown in numerous patients with Lynch syndrome. In the *BRCA2* gene, a heterozygous nonsense variant, c.7558C>T (p.Arg2520*), was identified in the patient PMCRC7. It has been previously reported in the ClinVar database as a P variant (Variation ID: 52353). For the *ATM* gene, a heterozygous nonsense variant, c.3687C>G (p.Tyr1229*), was detected in the patient PMCRC21. This variant is not present in population databases and was classified as LP according to the ACMG criteria. 

In the patient PMCRC8, four variants in three genes (*MUTYH, ATM,* and *BMPR1A*) were identified. For the *MUTYH* gene, two heterozygous variants, c.545G>A (p.Arg182His) and c.674C>T (p.Ser225Phe), were detected. The p.Arg182His variant has been reported multiple times as pathogenic (ClinVar Variation ID: 182689) in many individuals affected with familial adenomatous polyposis (FAP) and CRC. The *MUTYH* variant (p.Ser225Phe) and another two genes (*ATM* and *BMPR1A*) variants were categorized as uncertain significance.

Variants of unknown significance

A total of eight VUS in eight genes (*APC, ATM, BMPR1A, MLH1, MSH2, MUTYH, STK11,* and *TP53*) were detected in five patients (Table 2). Patient PMCRC2 carried two gene variants, one in *APC* (c.(835_934)_(1408_1548)dup and another in *MSH2* (c.554C>G, p.Ser185Cys). For the *APC* gene variant, a heterozygous duplication (c.(835_934)_(1408_1548)dup) was detected by the MLPA analysis. This variant led to duplication of exon 10-11 and was not observed in the gnomAD database. The *MSH2* (c.554C>G) variant was predicted to be highly deleterious by *in silico* prediction tools. Prediction by HOPE demonstrated that the serine residue 185 of DNA mismatch repair protein MSH2 is not evolutionarily conserved. The Ser185 is located in a domain that is important for the binding of other molecules and in contact with residues in a domain that is also important for binding. The p.Ser185Cys variant might disturb the interaction between these two domains and as such affect the function of the protein. Moreover, The Ser185 residue forms a hydrogen bond with Asp at position 181. The hydrophobicity difference between the serine and cysteine residues may affect the hydrogen-bond formation leading to the loss of hydrogen bonds in the core of the protein and as a result, disturb correct folding. Although the prediction tool suggests a deleterious effect of this variant, there is a lack of functional studies and/or animal models to confirm the above findings. According to all of the following information, both variants were classified as VUS.

As mentioned earlier, patient PMCRC8 harbors four variants in three genes (*MUTYH, ATM,* and *BMPR1A*). A heterozygous variant, p.Arg182His in the *MUTYH*, was pathogenic, while another heterozygous variant, p.Ser225Phe in the *MUTYH*, has never been reported before in any database. Results from *in silico* prediction tools suggested that the p.Ser225Phe was deleterious. However, the supporting evidence is currently insufficient to ascertain the role of this variant in the disease. Therefore, this variant has to be classified as VUS per the ACMG criteria. Furthermore, heterozygous missense variants, c.743G>A (p.Arg248Gln) and c.116C>T (p.Ser39Phe), were observed in *ATM* and *BMPR1A* genes, respectively. These two variants are reported in ClinVar as uncertain significance, Variation ID: 479015 and 230945, respectively.

The *MLH1* variant, c.1549G>A (p.Gly517Arg), was identified in the PMCRC9 patient. The *in silico* prediction tools reported conflicting results regarding the pathogenicity of this variant. This substitution is absent from gnomAD and has a single submission in the ClinVar database (variation ID 1404870) as uncertain significance. Therefore, the p.Gly517Arg variant was classified as VUS according to the evidence.

The *STK11* variant, c.1168G>A (p.Val390Met), was detected in the PMCRC1 patient. Many prediction tools predicted the benign consequence of the variant on protein structure and function. This variant is reported in dbSNP (rs374078532) and gnomAD exomes East Asian population database (allele frequency = 0.000113). Clinically significant assessments submitted by seven clinical laboratories in ClinVar were classified this variant as uncertain significance (variation ID: 142283). From all supporting data, the p.Val390Met variant was classified as VUS.

The *TP53* variant, c.304A>T (p.Thr102Ser), was observed in the PMCRC5 patient. Computational prediction tools are inconclusive regarding the impact of this missense variant on protein structure and function. Although the threonine residue 102 of cellular tumor antigen p53 is not evolutionarily conserved, the results from HOPE prediction showed that Thr102 is located within a stretch of residues annotated in UniProt as a special region (interaction with CCAR2). Substitution of p.Thr102Ser may disturb this region and its function. This variant is absent in the gnomAD cohort. ClinVar contains an entry for this variant as uncertain significance (variation ID: 582041) with four submissions. The evidence currently available is not sufficient to investigate the role of p.Thr102Ser in disease. Therefore, the clinical significance of this variant is uncertain. 

## Discussion

In this study, cancer-related molecular diagnostics testing in 36 genes by NGS was performed in 21 unrelated subjects with CRC. Most of the patients were diagnosed with rectum cancer (12 cases) (Table 1). Variant analysis revealed the presence of clinically significant P or LP variants in eight patients (38.1%), while a VUS was detected in four of the cases (19.1%).

The patient PMCRC2 was diagnosed with sigmoid colon cancer with FAP at the age of 29 years and had a strong family history of CRC (Figure 1). This patient carried two gene variants (exon 10-11 duplications in *APC *and missense in *MSH2*) which classified as VUS (Table 2). From the previous research about the *APC *gene, two duplications were detected in the FAP family [15,16]. The first one led to the duplication of exon 4 whereas the second one was a duplication of exons 10-11. These two duplication variants result in a frameshift and truncation of the APC protein. Based on this data, the duplication of exon 10-11 of the *APC* gene which was found in the patient PMCRC2 is assumed to be deleterious. The protein-truncation testing is required for the detection of abnormal proteins. As well as another variant, p.Ser185Cys in the *MSH2* gene, cosegregation analysis and further in-depth functional study of this variant are needed for understanding the disease mechanism. The results from all of the testing are useful for reclassification of the clinical significance of these two variants in the patient PMCRC2 that can change from VUS to P or LB/B.

The PMCRC7 patient developed ascending colon at the age of 33 years and had a family history of CRC, lymphoma, and uterine cancer (Figure 1). This patient harboring a pathogenic *BRCA2* variant, p.Arg2520* (ClinVar Variation ID: 52353), which has been observed in several individuals with hereditary breast-ovarian cancer (HBOC), prostate, pancreatic, esophageal and renal cancer from diverse ethnic origins. Although there is still controversy about the reported association of CRC with the *BRCA2* gene [17], the deleterious *BRCA2*-related CRC has also been observed in numerous studies [7,8,18,19]. Therefore, the pathogenic variant, p.Arg2520*, that was observed in the patient PMCRC7 was classified as a secondary finding. Unfortunately due to the scarcity of DNA samples from family members, it is challenging to explain the segregation of p.Arg2520* in this family. Recently, this patient was 44 years old. She underwent a mammogram and ultrasound, which revealed several cysts on right side and a few cysts on the left-side breast. The result was interpreted as negative (BIRADS2). Based on the available data, no association with HBOC was found in the current age of the patient. Although the pathogenic variant (p.Arg2520*) can be seen to be associated with cancer in multiple organs, it has never been reported in the colorectal. Therefore, this is the first report showing the presence of p.Arg2520* variant in CRC patients.

The PMCRC8, a female-sporadic patient diagnosed at 32, presented with rectum cancer. Colonoscopy disclosed a few polyps (less than 10 polyps) in the sigmoid colon. Variant analysis revealed two heterozygous variants (known pathogenic and unclassified variants) in *MUTYH *and a single heterozygous with VUS in *ATM* and *BMPR1A* (Table 2). *MUTYH*-associated polyposis (MAP) is an autosomal recessive inherited disorder. Biallelic germline variants in *MUTYH* lead to MAP and a tendency to develop CRC. Many different phenotypes, including classic and attenuated polyposis, can be seen in individuals with MAP. A previous study has shown that carriers with monoallelic have an elevated risk for CRC when compared with the general population [20]. Due to a lack of DNA samples from additional family members make it impossible to ascertain whether the *MUTYH* variants are in cis or trans. However, first-degree relatives should be screened for the same pathogenic variant to assess their risk of developing CRC. In addition, the absence of family members resulted in the inability to explain the *ATM* and *BMPR1A* gene variants as possible modifier genes or as possible causes of phenotypic variation within the patient's family. Therefore, further cosegregation analysis is essential to evaluate the disease penetrance of these three gene variants.

The PMCRC21, a 36-year-old male at the time of diagnosis, presented with transverse colon malignant neoplasm, and had a strong family history of CRC (Figure 1). Genetic testing revealed a nonsense variant (p.Tyr1229*) in the *ATM* gene. The deleterious variants in *ATM* have been demonstrated that it is a moderate penetrance gene correlated with increased susceptibility to breast cancer [21]. Furthermore, *ATM* has also been reported to be associated with the risk of CRC [6-8,10,11,19].

Our results are consistent with several published research reports, showing evidence of *APC* being the most frequently mutated gene detected in early-onset CRC patients [19,22,23]. Further analysis of the immunohistochemical staining of the four main DNA mismatch-repair (MMR) genes, the microsatellite instability (MSI) testing, or *MLH1* hypermethylation analysis, is required to confirm the diagnosis of the PMCRC9 patient. For individuals with a negative genetic test result, they may have causative variants in these genes that have yet to be discovered due to the limitations of sequencing or probably in other cancer susceptibility genes. Additionally, these patients may be suffering from sporadic cancer as a result of somatic mosaicism of the *APC* pathogenic variant [24], somatic hypermethylation of the *MLH1* gene [25], germline hypermethylation of *MSH2* (via 3' end deletions in the *EPCAM* gene) [26], or confluence of genetic and environmental risk factors.

## Conclusions

From this study, the causative gene was detected in eight out of 21 patients (38%) under 50 years of age. Our study demonstrated the benefits of using multigene cancer panel sequencing as an important tool for germline testing. Furthermore, the multigene panel also increases the possibility of detecting actionable secondary effects in other cancer-predisposing genes. Therefore, this method should be considered for screening in all individuals under 50 years with CRC. However, even though multi-gene testing increases the chances of detecting the deleterious gene, it also comes with management and treatment challenges, especially in patients harboring disease-causing variants in genes for which targeted therapies are not yet available.

## References

[1] Sung H, Ferlay J, Siegel RL, Laversanne M, Soerjomataram I, Jemal A, Bray F (2021). Global cancer statistics 2020: GLOBOCAN estimates of incidence and mortality worldwide for 36 cancers in 185 countries. CA Cancer J Clin.

[2] Sawicki T, Ruszkowska M, Danielewicz A, Niedźwiedzka E, Arłukowicz T, Przybyłowicz KE (2021). A review of colorectal cancer in terms of epidemiology, risk factors, development, symptoms and diagnosis. Cancers (Basel).

[3] (2023). Cancer in Thailand Vol X, 2016-2018. https://www.nci.go.th/e_book/cit_x/index.html.

[4] (2023). Public Health Statistics A.D. http://dmsic.moph.go.th/index/detail/9127.

[5] Nagahashi M, Wakai T, Shimada Y (2016). Genomic landscape of colorectal cancer in Japan: clinical implications of comprehensive genomic sequencing for precision medicine. Genome Med.

[6] Yurgelun MB, Kulke MH, Fuchs CS (2017). Cancer susceptibility gene mutations in individuals with colorectal cancer. J Clin Oncol.

[7] Hansen MF, Johansen J, Sylvander AE (2017). Use of multigene-panel identifies pathogenic variants in several CRC-predisposing genes in patients previously tested for Lynch Syndrome. Clin Genet.

[8] Toh MR, Chiang JB, Chong ST (2018). Germline pathogenic variants in homologous recombination and DNA repair genes in an Asian cohort of young-onset colorectal cancer. JNCI Cancer Spectr.

[9] Olkinuora AP, Peltomäki PT, Aaltonen LA, Rajamäki K (2021). From APC to the genetics of hereditary and familial colon cancer syndromes. Hum Mol Genet.

[10] Fujita M, Liu X, Iwasaki Y (2022). Population-based screening for hereditary colorectal cancer variants in Japan. Clin Gastroenterol Hepatol.

[11] Jiang W, Li L, Ke CF (2022). Universal germline testing among patients with colorectal cancer: clinical actionability and optimised panel. J Med Genet.

[12] Lertwilaiwittaya P, Roothumnong E, Nakthong P (2021). Thai patients who fulfilled NCCN criteria for breast/ovarian cancer genetic assessment demonstrated high prevalence of germline mutations in cancer susceptibility genes: implication to Asian population testing. Breast Cancer Res Treat.

[13] Richards S, Aziz N, Bale S (2015). Standards and guidelines for the interpretation of sequence variants: a joint consensus recommendation of the American College of Medical Genetics and Genomics and the Association for Molecular Pathology. Genet Med.

[14] Venselaar H, Te Beek TA, Kuipers RK, Hekkelman ML, Vriend G (2010). Protein structure analysis of mutations causing inheritable diseases. An e-Science approach with life scientist friendly interfaces. BMC Bioinform.

[15] McCart A, Latchford A, Volikos E, Rowan A, Tomlinson I, Silver A (2006). A novel exon duplication event leading to a truncating germ-line mutation of the APC gene in a familial adenomatous polyposis family. Fam Cancer.

[16] Kaufmann A, Vogt S, Uhlhaas S (2009). Analysis of rare APC variants at the mRNA level: six pathogenic mutations and literature review. J Mol Diagn.

[17] Sopik V, Phelan C, Cybulski C, Narod SA (2015). BRCA1 and BRCA2 mutations and the risk for colorectal cancer. Clin Genet.

[18] Garre P, Martín L, Sanz J (2015). BRCA2 gene: a candidate for clinical testing in familial colorectal cancer type X. Clin Genet.

[19] Valle L, Vilar E, Tavtigian SV, Stoffel EM (2019). Genetic predisposition to colorectal cancer: syndromes, genes, classification of genetic variants and implications for precision medicine. J Pathol.

[20] Win AK, Dowty JG, Cleary SP (2014). Risk of colorectal cancer for carriers of mutations in MUTYH, with and without a family history of cancer. Gastroenterology.

[21] Shiovitz S, Korde LA (2015). Genetics of breast cancer: a topic in evolution. Ann Oncol.

[22] Almuzzaini B, Alghamdi J, Alomani A (2021). Identification of novel mutations in colorectal cancer patients using AmpliSeq comprehensive cancer panel. J Pers Med.

[23] Dos Santos W, Sobanski T, de Carvalho AC (2019). Mutation profiling of cancer drivers in Brazilian colorectal cancer. Sci Rep.

[24] Spier I, Drichel D, Kerick M (2016). Low-level APC mutational mosaicism is the underlying cause in a substantial fraction of unexplained colorectal adenomatous polyposis cases. J Med Genet.

[25] Kuismanen SA, Holmberg MT, Salovaara R, de la Chapelle A, Peltomäki P (2000). Genetic and epigenetic modification of MLH1 accounts for a major share of microsatellite-unstable colorectal cancers. Am J Pathol.

[26] Kuiper RP, Vissers LELM, Venkatachalam R (2011). Recurrence and variability of germline EPCAM deletions in Lynch syndrome. Human Mutation.

